# Identification and functional assessment of a *KCNH2* compound heterozygosity in a patient with presumed idiopathic ventricular fibrillation ascertains the diagnosis of long QT syndrome type 2

**DOI:** 10.1093/europace/euag001

**Published:** 2026-01-09

**Authors:** Natálie Janková, Martin Král, Olga Švecová, Jana Zídková, Samuel Lietava, Stanislava Sladeček, Jiří Pacherník, Michal Pásek, Tomáš Novotný, Markéta Bébarová

**Affiliations:** Department of Physiology, Faculty of Medicine, Masaryk University, Kamenice 5, Brno 625 00, Czech Republic; Department of Physiology, Faculty of Medicine, Masaryk University, Kamenice 5, Brno 625 00, Czech Republic; Department of Physiology, Faculty of Medicine, Masaryk University, Kamenice 5, Brno 625 00, Czech Republic; Department of Internal Medicine, Center of Molecular Biology and Genetics, Hematology and Oncology, University Hospital Brno and Faculty of Medicine, Masaryk University, Jihlavská 20, Brno 625 00, Czech Republic; Department of Internal Medicine and Cardiology, University Hospital Brno and Faculty of Medicine, Masaryk University, Jihlavská 20, Brno 625 00, Czech Republic; Department of Experimental Biology, Faculty of Science, Masaryk University, Kamenice 5, Brno 625 00, Czech Republic; Department of Experimental Biology, Faculty of Science, Masaryk University, Kamenice 5, Brno 625 00, Czech Republic; Department of Physiology, Faculty of Medicine, Masaryk University, Kamenice 5, Brno 625 00, Czech Republic; Institute of Thermomechanics, Czech Academy of Sciences, Dolejškova 5, Prague 182 00, Czech Republic; Department of Internal Medicine and Cardiology, University Hospital Brno and Faculty of Medicine, Masaryk University, Jihlavská 20, Brno 625 00, Czech Republic; Department of Physiology, Faculty of Medicine, Masaryk University, Kamenice 5, Brno 625 00, Czech Republic; Department of Internal Medicine and Cardiology, University Hospital Brno and Faculty of Medicine, Masaryk University, Jihlavská 20, Brno 625 00, Czech Republic

**Keywords:** hERG, Idiopathic ventricular fibrillation, LQT2, Expression, Patch clamp, Mathematical modelling

## Abstract

**Background and Aims:**

The *KCNH2* (*hERG*) gene encodes the Kv11.1 protein, the pore-forming subunit of the rapid delayed rectifier potassium channel, which plays a key role in cardiac repolarization. We aimed to investigate the function of two Kv11.1 variants *in trans*, S1021Qfs*98 and A228V, identified in a patient suffering from idiopathic ventricular fibrillation (VF).

**Methods:**

A detailed clinical and genetic investigation was followed by functional analysis using the whole-cell patch clamp technique, western blot, and mathematical simulations in a human ventricular cell model.

**Results:**

In comparison with wild type, the current was decreased by 69.5 and 69.2% in S1021Qfs*98 and S1021Qfs*98/A228V, respectively, which agreed well with a significant decrease in the expression of S1021Qfs*98 channels, but no differences were observed in A228V. The voltage dependence of activation and inactivation and the time course of activation and deactivation remained unchanged. Minor changes were observed in the time course of inactivation and recovery from inactivation in S1021Qfs*98 and S1021Qfs*98/A228V. Arrhythmogenesis based on early afterdepolarizations (EADs) at rest, provoked by hypokalemia, and during β-adrenergic stimulation was suggested by simulations in a human ventricular cell model.

**Conclusion:**

To conclude, A228V is a benign variant, whereas S1021Qfs*98 exhibits a loss-of-function defect and dominant negativity. EADs-related arrhythmogenesis was predicted, which explains the pathogenic phenotype of the proband carrying both these variants and experiencing repetitive VF episodes. Based on the findings, we reclassify S1021Qfs*98 as a pathogenic, LQT2-associated variant. The data highlight the importance of functional analysis for the correct management of patients with idiopathic VF and genetic variants.

## Graphical Abstract

**Graphical Abstract:**
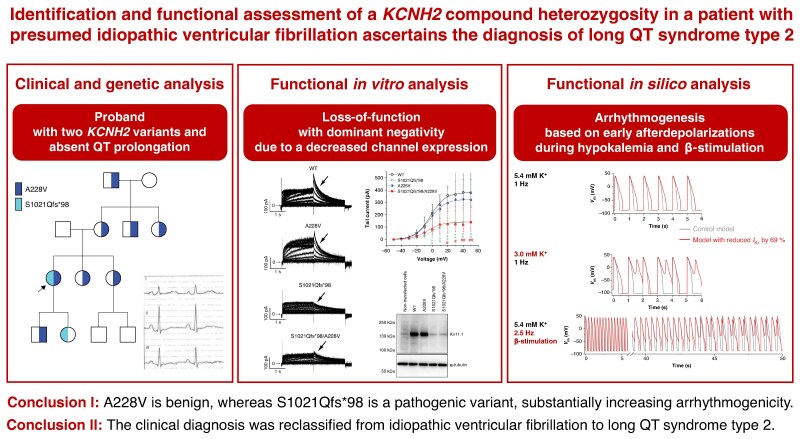

## Introduction

The *KCNH2* gene, also known as *hERG* (*human ether-a-go-go related gene*), encodes the Kv11.1 protein, which serves as a pore-forming subunit of the voltage-gated channel responsible for the rapid delayed rectifier potassium current (*I*_Kr_), playing an essential role in cardiac repolarization. Variants in the *KCNH2* gene may interfere with the expression and stability of the Kv11.1 protein, its trafficking to the cell surface, gating of the channel, or the channel’s permeability.^1^ Dysfunction of Kv11.1 influences repolarization and, thus, the duration of the QT interval on the electrocardiogram (ECG). Clinical manifestations of this state include inherited arrhythmogenic syndromes, namely long QT syndrome (LQTS) type 2 (LQT2), characterized by a loss of channel function and consequent delay in cardiac repolarization.^2^

In some patients, an episode of sudden cardiac arrest due to ventricular fibrillation (VF) cannot be explained by apparent structural or functional myocardial disease^3^ or by extracardiac aetiologies, including pulmonary, metabolic, or toxicological causes.^4^ Under these circumstances, a diagnosis of idiopathic VF is made. Genetic testing may uncover variants in genes encoding cardiac ion channels and associated proteins in some of these patients. Most idiopathic VF cases are linked to variants of uncertain significance (VUS),^5^ in which the impact on ion channel dysfunction cannot be predicted using standard software tools. The functional analysis is then the only way to assess the pathogenic character of the variant. To date, several VUS in the *KCNH2* gene not clearly related to congenital LQT2 have been identified and functionally characterized, namely S906L^6^ and P347S.^7^

In this study, we provide clinical, genetic, and functional *in vitro* and *in silico* characterization of a proband suffering from idiopathic VF and carrying two variants in the *KCNH2* gene, S1021Qfs*98 and A228V. The new data revealed the arrhythmogenic potential of the S1021Qfs*98 variant and explained arrhythmogenesis in the proband, leading to reclassification as a LQT2 pathogenic variant.

## Methods

The study adhered to the principles outlined in the Declaration of Helsinki and was approved by the Multicenter Ethical Committee, University Hospital Brno (Brno, Czech Republic). All participants signed a written informed consent form. In participants under the age of 18 years, written informed consent was obtained from a parent and/or legal guardian.

Diagnosis of idiopathic VF was established according to ESC Guidelines.^8,9^

### Clinical examination

During the index hospitalization, a physical examination, blood tests, information on medical history, 2D transthoracic echocardiography (2D-TTE), baseline 12-lead ECG, and continuous ECG monitoring were obtained. 2D-TTE findings were considered normal if the ejection fraction of the left ventricle was ≥50% (calculated using the Teichholz and Simpson formula) and in the presence of a maximally mild valvular disease. ECGs recorded during index hospitalization were reviewed by at least two cardiologists. The ECG recordings were printed at a paper speed of 25 mm/s and an amplitude of 10 mm/mV. A diagnosis of Brugada syndrome was established if a coved ST-segment elevation ≥2 mm was documented spontaneously in at least one lead from V1–2 positioned in the 4th, 3rd, and 2nd intercostal space. LQTS was suspected if the QT interval on the baseline ECG, corrected to the heart rate using Fridericia’s and Bazett’s formulas, was ≥480 ms. A bicycle exercise stress test was performed, starting at 0.5 W/kg and increasing by 0.5 W/kg every 3 min to achieve 75–100% of the age-specific maximal heart rate, which was defined as 220 beats per minute minus age in years (12-lead ECG with standard Mason–Likar electrode positions; 50 mm/s, 20 mm/mV). LQTS was diagnosed if a repeated QT interval prolongation (QTc ≥480 ms) was observed or the diagnostic score (the so-called Schwartz score^10,11^) was >3. Catecholamine polymorphic ventricular tachycardia was diagnosed if bidirectional or polymorphic ventricular tachycardia was provoked during the exercise stress test. An electrophysiology study was performed and conduction intervals were measured. A programmed ventricular stimulation was performed from the apex and the outflow tract of the right ventricle. The protocol included: (i) a stimulation frequency of 120/min with extra stimuli of the cycle length (CL) 280/250/200 ms, a pacing frequency of 140/min with extra stimuli of CL 220/250/300 ms and bursts of 250 ms 1:1 and 300 ms 2:1 from the apex of the right ventricle, and (ii) a stimulation frequency of 120/min with extra stimuli of CL 240/200/200 ms, stimulation frequency 140/min with extra stimuli of CL 220/200/200 ms, burst 250 ms 2:1 from the outflow tract of the right ventricle.

### Genetic analysis

For the identification of sequence variants in arrhythmogenic genes, we used the capture method KAPA HyperChoice (Roche, USA) followed by next-generation sequencing on the NextSeq 500 (Illumina, USA). Identified variants were classified based on the ACMG guidelines^12^ and further evaluated using the VarSome variant classifier (https://varsome.com). Segregation analysis was performed by PCR and Sanger sequencing with the BigDye Terminator Cycle Sequencing Kit (Applied Biosystems, USA) on the ABI 3130xl Genetic Analyzer (Applied Biosystems, USA).

### Biophysical analysis

Experiments were conducted on Chinese hamster ovary (CHO) cell line (ECACC collection; Merck KGaA, Darmstadt, Germany) cultured at 37°C/5% CO_2_ in the Ham’s F-12 medium supplemented with 10% foetal bovine serum, 0.005% gentamycin, and 2 mM L-glutamine (Sigma-Aldrich, St. Louis, MO, USA).

The cells were transfected with the *KCNH2* gene plasmids (kindly provided as a gift by prof. Gail Robertson, University of Wisconsin-Madison, USA), containing either wild-type (WT) or mutated human *KCNH2* in a pcDNA3 vector, which were isolated from *Escherichia coli* using the endotoxin-free QIAprep Spin Miniprep Kit (Qiagen, Hilden, Germany). The amount of 0.7 µg of *KCNH2* plasmid per Petri dish was co-transfected with 0.4 µg of the plasmid encoding the green fluorescent protein (GFP; pIRES2-eGFP vector kindly provided as a gift by prof. Paul G. A. Volders, Maastricht University, The Netherlands) to identify cells expressing the transfected plasmids. The variants c.3060dup p.(S1021Qfs*98) and c.683C>T p.(A228V) in the human *KCNH2* were constructed using site-directed mutagenesis using QuikChange II XL Site-Directed Mutagenesis Kit (Agilent Technologies, Cedar Creek, TX, USA) and VectorBuilder service (https://vectorbuilder.com), respectively. TransFast Transfection Reagent (Promega, Madison, WI, USA) was used for the transfection 24 h after seeding of CHO cells into Petri dishes. Five populations of the cells were generated and used: (i) cells carrying S1021Qfs*98-Kv11.1 variant; (ii) cells carrying A228V-Kv11.1 variant; (iii) cells carrying both variants A228V-Kv11.1 and S1021Qfs*98-Kv11.1 (ratio 1:1) to simulate the compound heterozygous state in the proband; (iv) cells carrying WT-Kv11.1 (the control group); and (v) non-transfected cells (the negative control group).

After 24 h, cells were gently harvested from the culture dish using trypsin and washed away with the supplemented Ham’s medium (for details, see above). They were then kept at room temperature before undergoing electrophysiological measurement. The measurement was performed by the whole-cell patch clamp technique (in the voltage-clamp mode) at 37°C using the Axopatch 200B amplifier, Digidata 1440A, and pCLAMP 9.2 software (Molecular Devices, Sunnyvale, CA, USA). The resistance of filled borosilicate glass electrodes pulled and heat-polished using a programmable horizontal puller (Zeitz-Instrumente Vertriebs GmbH, Martinsried, Germany) was kept below 2.5 MΩ; the series resistance was compensated up to 60%. The measured cells were held at a potential of −80 mV; for stimulation protocols and stimulation frequencies, see Results.

Measured cells were washed with Tyrode solution (in mM: NaCl 132, KCl 4.8, CaCl_2_ 2.0, MgCl_2_ 1.2, HEPES 10, and glucose 5; pH 7.4, adjusted with NaOH). The pipette solution was composed of (in mM): K-aspartate 110, K_2_ATP 5, CaCl_2_ 1, MgCl_2_ 1, EGTA 11, and HEPES 10 mM (pH 7.3, adjusted with KOH). The junction potential was ∼15 mV (calculated using Clampex). The chemicals were purchased from Sigma-Aldrich (Prague, Czech Republic), unless otherwise indicated.

The data were analysed and fitted using the software Clampfit 10.3 (Molecular Devices) and Origin 2022b (OriginLab Corporation). To assess the voltage dependence of the current during activation and inactivation, fitting of the data was performed using the Boltzmann equation as follows:


$$y=\frac{A_{1}-\;A_{2}}{1+\;e^{\frac{\left(V-\;V_{1/2}\right)}{k}}}+A_{2},$$

where *A*_1_ is the minimal and *A*_2_ the maximal value, *V* is the membrane voltage, *V*_1/2_ is the voltage of half-maximal activation or inactivation, and *k* is the slope factor.

Analysis of the cell membrane capacitance *C*_m_ and access resistance *R*_a_ was performed by using an in-house MATLAB script available on GitHub, (https://github.com/rguide).

### Western blot analysis

CHO cells were directly lysed in Laemmli buffer [100 mM Tris/HCl (pH 6.8), 20% glycerol, 1% SDS, 0.01% bromophenol blue, and 1% 2-mercaptoethanol] 24 h after the transfection. Western blotting was performed according to the manufacturer’s instructions with minor modifications [SDS-PAGE run at 100 V, transfer onto PVDF membrane for 1 h at 115V (Bio-Rad)]. Membranes were blocked in 5% non-fat dry milk solution in TBS-T [composition: 10 mM Tris/HCl (pH 7.6), 100 mM NaCl, and 0.08% Tween-20] for 30 min and subsequently incubated overnight at 4°C with primary antibodies Kv11.1 (ThermoFisher OSK00026W, dilution 1:1000) and α-tubulin (Cell Signaling, cs-5335, dilution 1:2000). Next, membranes were washed in TBS-T and incubated with HRP-conjugated secondary antibodies (Sigma-Aldrich). Immunoreactive bands were detected using ECL detection reagent kit (Merck-Millipore) and the FusionSL chemiluminescence documentation system (Vilber-Lourmat). Results were quantified by the densitometric analysis of western blot bands using the Fiji distribution of ImageJ.

### Mathematical modelling

To simulate the impact of the studied dysfunction in *I*_Kr_ channels on the electrical activity and Ca²⁺ transients (CaT) in human ventricular cardiomyocytes, we utilized the previously published ORd model of O’Hara *et al.*,^13^ which was configured for a midmyocardial cell. The only modification we introduced to the original model was a 20% reduction in the Ca²⁺ permeability of L-type Ca²⁺ channels (*P*_Ca_), which ensured that the model reconstruction of the experiment performed by Li *et al.*^14^ on human midmyocardial cells yielded an action potential duration at 90% repolarization (APD_90_) within the experimentally observed range (390 ± 30 ms).

To simulate the arrhythmogenic effects induced by β-adrenergic stimulation (β-AS) with 1 µM isoproterenol, we adopted the β-AS formulation described in the supplement of the study by O’Hara and Rudy.^15^ However, the originally proposed hyperpolarizing shift of the steady-state activation curve of L-type Ca²⁺ current (*I*_Ca_) by −16 mV was reduced to −3 mV, consistent with the shift of −2.8 mV applied to the steady-state inactivation curve. This modification was necessary to prevent spontaneous early afterdepolarizations (EADs) that were observed in the midmyocardial configuration of the ORd model with the original β-AS-induced −16 mV shift (due to a markedly increased *I*_Ca_ window current), even in the absence of *I*_Kr_ dysfunction (i.e. in the control model), which is inconsistent with experimental observations in non-failing human ventricular myocardium during β-AS. Reducing the activation shift to −3 mV preserved the characteristic β-adrenergic enhancement of *I*_Ca_ while maintaining the electrical stability of the control midmyocardial cell model.

### Statistical analysis

The statistical analysis was performed using the software GraphPad Prism, version 9.5.1 (GraphPad Software, Inc.); *P* < 0.05 was considered statistically significant. Only significances in relation to WT are marked in all graphs; for other significances, see *Table 1*.

**Table 1:** Biophysical characteristics of WT, S1021Qfs*98, A228V, and S1021Qfs*98/A228V channels

	WT	S1021Qfs*98	A228V	S1021Qfs*98/A228V
**Basic characteristics**	*n* = 15	*n* = 14	*n* = 13	*n* = 14
*C*_m_ (pF)	13.6 (Q1 12.7, Q3 18.0)	19.4 (Q1 9.9, Q3 25.5)	15.3 (Q1 10.7, Q3 24.1)	17.8 (Q1 9.5, Q3 24.7)
*R*_a_ (MΩ)	4.24 (Q1 3.60, Q3 6.30)	3.44 (Q1 2.99, Q3 4.98)	3.52 (Q1 2.90, Q3 4.98)	4.42 (Q1 2.86, Q3 6.29)
				
**Activation**	*n* = 15	*n* = 14	*n* = 13	*n* = 14
*I*_tail_ at +30 mV (pA)	362.7 (Q1 231.2, Q3 489.2)	110.5 (Q1 58.3, Q3 157.2)**	322.9 (Q1 262.5, Q3 530.7)^$$,¥^	111.8 (Q1 62.9, Q3 229.2)^#^
*V*_1/2_ (mV)	−2.76 (Q1 −10, Q3 12.48)	7.30 (Q1 2.69, Q3 14.73)	−5.79 (Q1 −9.77, Q3 3.34)	−5.5 (Q1 −11.54, Q3 −1.78)
*k*	8.50 (Q1 7.7, Q3 10.09)	9.58 (Q1 8.68, Q3 13.24)	9.63 (Q1 7.99, Q3 11.30)	8.54 (Q1 7.85, Q3 9.06)
	*n* = 15	*n* = 9	*n* = 13	*n* = 14
*τ*_act_ at +30 mV (ms)	164.4 (Q1 99.4, Q3 368.0)	107.3 (Q1 63.5, Q3 210.4)	186.4 (Q1 98.6, Q3 238.8)	112.8 (Q1 67.3, Q3 158.9)
				
**Deactivation**	*n* = 15	*n* = 15	*n* = 12	*n* = 9
*τ*_deact_ at −90 mV (ms)	122.1 (Q1 77.0, Q3 211.0)	154.1 (Q1 123.6, Q3 227.9)	125.3 (Q1 60.4, Q3 218.9)	188.0 (Q1 109.5, Q3 201.0)
				
**Inactivation**	*n* = 14	*n* = 10	*n* = 19	*n* = 17
*V*_1/2_ (mV)	−60.4 (Q1 −63.1, Q3 −52.2)	−54.2 (Q1 −71.2, Q3 −46.8)	−60.8 (Q1 −66.9, Q3 −48.6)	−64.9 (Q1 −68.6, Q3 −63.0)
*k*	23.1 (Q1 22.4, Q3 26.6)	27.4 (Q1 25.0, Q3 37.1)	27.0 (Q1 23.9, Q3 31.3)	24.4 (Q1 22.0, Q3 28.2)
	*n* = 14	*n* = 10	*n* = 17	*n* = 11
*τ*_inact_ at 0 mV (ms)	5.84 (Q1 3.64, Q3 7.08)	9.05 (Q1 7.90, Q3 10.67)^£££^	3.69 (Q1 3.21, Q3 4.40)^$$^	3.56 (Q1 2.63, Q3 4.73)
	*n* = 14	*n* = 10	*n* = 19	*n* = 15
τ_rec_ at −80 mV (ms)	1.38 (Q1 0.27, Q3 2.20)	0.46 (Q1 0.27, Q3 0.64)	1.39 (Q1 0.93, Q3 1.85)	0.97 (Q1 0.80, Q3 1.18)
τ_rec_ at −20 mV (ms)	4.63 (Q1 4.03, Q3 4.96)	1.99 (Q1 1.80, Q3 3.06)***	3.10 (Q1 2.47, Q3 3.34)^€€^	2.62 (Q1 2.25, Q3 3.17)^###^

Median values and the 1_st_ and 3_rd_ quartiles (Q1 and Q3, respectively) are listed; *C*_m_ - cell membrane capacitance, *R*_a_ - access resistance, *I*_tail_ - tail current, *V*_1/2_ - half-maximal activation/inactivation voltage, *k* - slope factor, *τ*_act_, *τ*_deact_, *τ*_inact_, and *τ*_rec_ - time constant of activation, deactivation, inactivation, and recovery from inactivation, respectively;

** and *** – *P* < 0.01 and 0.001, respectively, S1021Qfs*98 *vs.* WT.

^#^ and ^###^*P* < 0.05 and 0.001, respectively, S1021Qfs*98/A228V *vs.* WT.

^$$^ *P* < 0.01, S1021Qfs*98 *vs.* A228V.

^€€^ *P* < 0.01, A228V *vs.* WT.

^£££^ *P* < 0.001, S1021Qfs*98 *vs.* S1021Qfs*98/A228V.

^¥^ *P* < 0.05, A228V *vs.* S1021Qfs*98/A228V.

In the case of the biophysical data, the normal data distribution was rejected using the Shapiro–Wilk test.^16^ All data, including western blot, are presented as the median ± interquartile range (from *n* cells or samples). To determine the significance of differences among groups, the Kruskal–Wallis test for unpaired data with Dunn’s multiple comparison *post hoc* test was performed.

## Results

### Proband with two *KCNH2* variants *in trans* and diagnosis of idiopathic ventricular fibrillation

**Figure 1:**
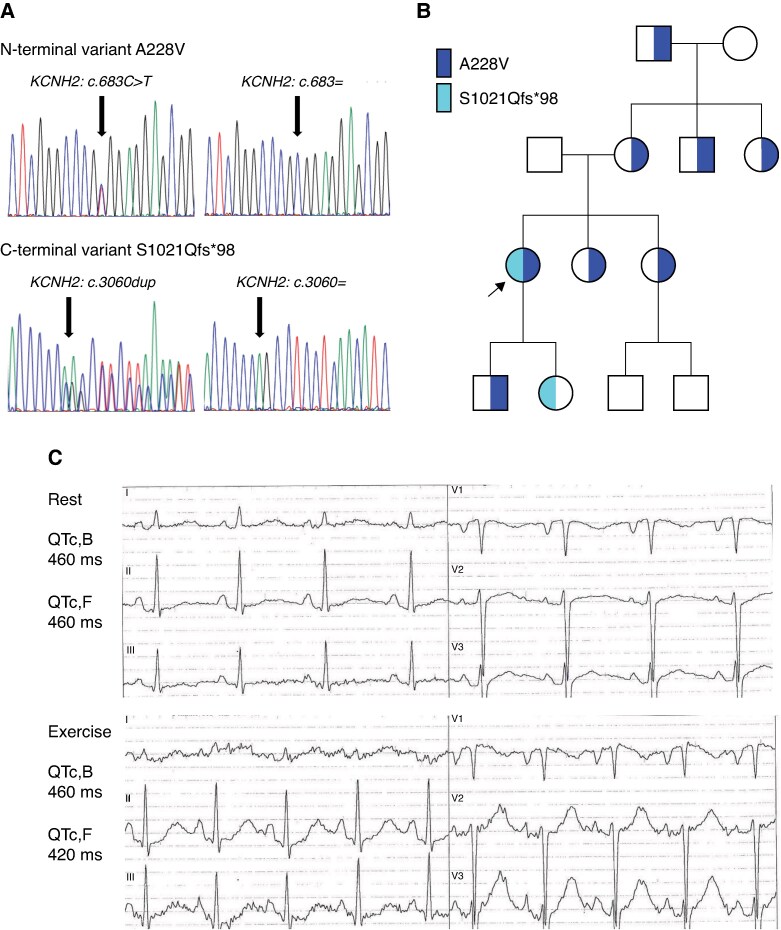
Clinical and genetic characteristics. (*A*) Sequence analysis of the revealed variants A228V and S1021Qfs*98. (*B*) Pedigree of the proband’s family; A228V in blue, S1021Qfs*98 in cyan. (*C*) ECG of the proband at rest (up) and at the 4th minute of recovery after the exercise test (down); 20 mm/mV, 50 mm/s. ECG, electrocardiogram.

The clinical and genetic characteristics of the proband and her relatives are illustrated in *Figure 1*. The proband is a female who experienced her first VF episode at the age of 22. After being successfully resuscitated, a detailed clinical examination was performed (including 2D-TTE, ECG at rest and during the exercise test, Holter monitoring, and basic biochemical parameters). No potential underlying pathology was detected; QTc,B and QTc,F were both 460 ms at rest and 460 and 420 ms, respectively, during recovery after the exercise test (*Figure 1C*). No arrhythmias were detected during or after the exercise test, nor during Holter monitoring. An electrophysiological study of the heart showed normal parameters of the conduction system; no supraventricular or ventricular tachycardia was induced; an accessory pathway was excluded. The patient refused the sodium channel blocker challenge. An implantable cardioverter defibrillator (ICD) was implanted as a secondary prevention of sudden cardiac death and treatment with the β-blocking agent betaxolol was started. Within the following 7-year follow-up period, 16 episodes of VF were documented, mostly during sleep (5 times with spontaneous termination, 5 VF episodes treated with ICD discharges) or hypokalemia (2.9 mM; 6 ICD discharges to interrupt VF due to an electric storm). Then, the proband and her family moved out and our follow-up ended. Recently, the proband has been contacted and she has reported a continuing trend of repeated VF episodes during sleep, but also when she was suddenly scared (e.g. alarm clock ringing), or during physical activity.

Using a panel of arrhythmogenic genes, two variants in the *KCNH2* (*hERG*, *NM_000238.4*) gene *in trans* were detected. The first one was a missense variant c.683C>T (*Figure 1A*, upper panel), which resulted in the amino acid change from alanine (A) to valine (V) at position 228, i.e. in the N-terminus of the Kv11.1 channel, and was predicted as benign (AlphaMissense) and classified as likely benign. This finding agrees with that of O'Neill *et al.*,^17^ who demonstrated (by performing a multiplexed assay of variant effect) that the A228V variant is capable of trafficking to the cell surface, which supports the premise of its benign potential. The A228V variant was also detected in seven asymptomatic relatives of the proband (*Figure 1B*). The second variant was duplication c.3060dup (*Figure 1A*, bottom panel), which resulted in an amino acid change from serin (S) at position 1021 to glutamine (Q), i.e. in the C-terminus of Kv11.1 channel, and formation of a premature stop codon 98 amino acids later (S1021Qfs*98; missing 42 amino acids at the end of the channel). This seems to be a *de novo* variant since both parents were negative for this variant (*Figure 1B*). The only family member positive for the S1021Qfs*98 variant was the daughter of the proband. We have only limited information on her clinical status, but we know that a prolonged QTc interval during sleep was detected and that she was treated with a β-blocker. This frame-shift variant results in a premature termination codon at position 1118, located within the last exon. Although the ACMG criterion PVS1 cannot be applied at very strong strength for this variant,^18^ it remains classified as likely pathogenic when combined with other supporting evidence criteria.

To sum up, we identified a female proband suffering from repeated episodes of VF and with two *KCNH2* variants *in trans*, one of them being predicted to be likely pathogenic. However, the QTc interval was within the physiological range. Thus, a diagnosis of idiopathic VF was made.

### S1021Qfs*98 as well as S1021Qfs*98/A228V channels showed substantially decreased and comparable current

**Figure 2:**
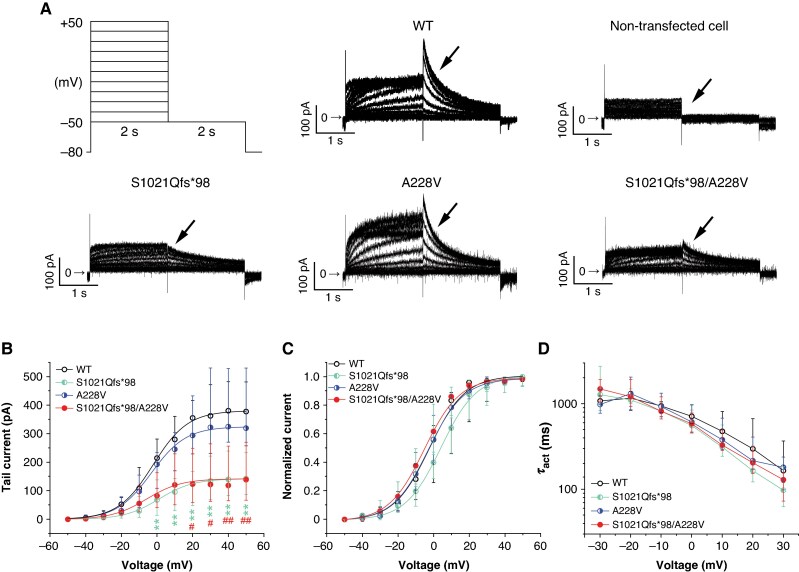
Impact of S1021Qfs*98- and A228V-hERG variants on channel activation. (*A*) Scheme of the stimulation protocol (left upper panel) and representative traces in all cell groups, namely WT, S1021Qfs*98, A228V, and S1021Qfs*98/A228V cells (*n* = 15, 14, 13, and 14, respectively). No current was detected in non-transfected cells (*n* = 19). (*B*) Average voltage dependence of tail current activation; **P < 0.01 for S1021Qfs*98 vs. WT, and ^#^ and ^##^*P* < 0.05 and 0.01, respectively, for S1021Qfs*98/A228V vs. WT (Kruskal–Wallis test with Dunn’s post-test; only significance vs. WT is shown, for others, see *Table 1*). (*C*) Voltage dependence of steady-state activation. (*D*) Voltage dependence of the time constant of activation (τ_act_); *n* = 15, 9, 13, and 14 cells for WT, S1021Qfs*98, A228V, and S1021Qfs*98/A228V, respectively. WT, wild type.

To investigate activation of the Kv11.1 channel, we applied a protocol consisting of two 2-s impulses, the first one depolarizing the cell from the holding voltage of −80 mV to voltages between −50 and +50 mV (10-mV steps) and the second one repolarizing the membrane voltage to −50 mV (*Figure 2A*, left upper panel; the stimulation frequency was 0.2 Hz). Typical Kv11.1 current was detected in WT channels (for a representative trace, see *Figure 2A*, middle upper panel), but no measurable current was present in non-transfected cells, as expected (for a representative trace, see *Figure 2A*, right upper panel; *n* = 19).

Since a positive correlation between the current amplitude and *C*_m_ was not observed in any tested cell groups (see Supplementary material online, *Figure S1*), conversion of the current amplitude to the current density was not performed (*Figure 2B*), in accordance with the recommendations of Kula *et al*.^19^ No significant differences were detected in *C*_m_ and *R*_a_ values among the groups (medians: *C*_m_ = 13.6 pF, *R*_a_ = 4.24 MΩ in WT, *n* = 15; *C*_m_ = 19.4 pF, *R*_a_ = 3.44 MΩ in S1021Qfs*98, *n* = 14; *C*_m_ = 15.3 pF, *R*_a_ = 3.52 MΩ in A228V, *n* = 13; *C*_m_ = 17.8, *R*_a_ = 4.42 MΩ in S1021Qfs*98/A228V, *n* = 14; *P* > 0.05 in all cases; Supplementary material online, *Figure S2*).

As apparent from the average data in *Figure 2B* and *Table 1*, the tail current (evaluated as the peak current at the beginning of the second pulse) was significantly decreased in both S1021Qfs*98 and S1021Qfs*98/A228V channels, namely by 69.5 and 69.2%, respectively (e.g. median at +30 mV: 110.5 pA in S1021Qfs*98, *n* = 14, **P < 0.01, and 111.8 pA in S1021Qfs*98/A228V, *n* = 14, ^#^*P* < 0.05, vs. 362.7 pA in WT, *n* = 15), but not in A228V channels (e.g. median at +30 mV: 322.9 pA in A228V, *n* = 13, *P* > 0.05 vs. WT; for other significances, see *Table 1*). After normalization to the respective maximal tail current, the voltage dependence of steady-state activation could be analysed using fitting with the Boltzmann equation (see Methods; *Figure 2C*). Although the curve for S1021Qfs*98 channels seemed to be slightly shifted to the right, no statistical significance was detected among the tested groups (for average values of the half-maximal activation voltage *V*_1/2_ and slope factor *k*, see *Table 1*).

Single exponential fitting of traces at −30 to +30 mV during the first impulse revealed that no significant differences were apparent in the time constant of the activation *τ*_act_ (e.g. median at +30 mV: 164.4 ms in WT, *n* = 15; 107.3 ms in S1021Qfs*98, *n* = 9; 186.4 ms in A228V, *n* = 13; 112.8 ms in S1021Qfs*98/A228V, *n* = 14; *Figure 2D*, *Table 1*).

**Figure 3:**
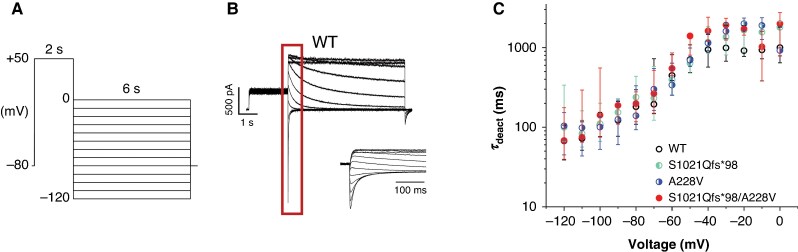
hERG channel deactivation was not significantly changed by S1021Qfs*98 and A228V variants. (*A*) Scheme of the stimulation protocol. (*B*) A representative recording in WT channels; inset: a detail view of the key part of the protocol (marked by the dark red rectangle). (*C*) Voltage dependence of the time constant of deactivation (τ_deact_) in WT, S1021Qfs*98, A228V, and S1021Qfs*98/A228V channels (*n* = 15, 15, 12, and 9, respectively). WT, wild type.

The time course of channel deactivation was explored using a stimulation protocol consisting of 2-s depolarizing prepulse from −80 to +50 mV followed by 6-s repolarizing impulse gradually decreasing from 0 mV to −120 mV (10-mV steps; the stimulation frequency was 0.25 Hz; for a scheme of the protocol, see *Figure 3A*); the time constant of deactivation (*τ*_deact_) was determined using a single exponential fit (for a representative trace in WT, see *Figure 3B*). No significant differences were observed among groups (for average values of *τ*_deact_, see *Figure 3C*, *Table 1*).

**Figure 4:**
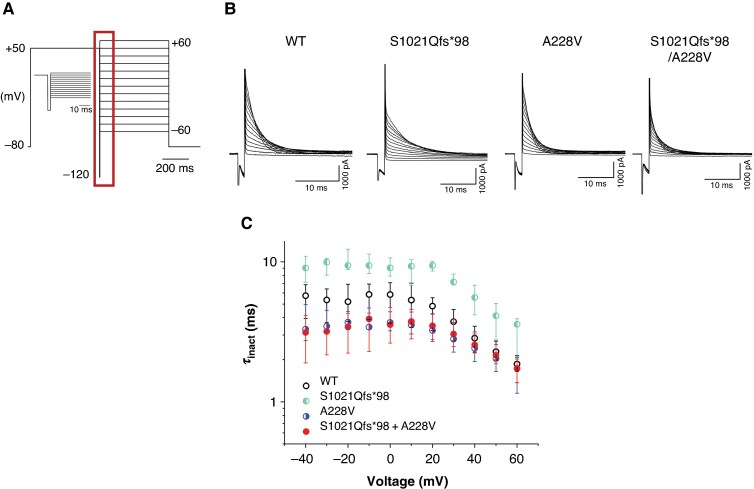
Time course of inactivation of Kv11.1 channels. (A) Scheme of experimental protocol; inset: a detailed view of the key part of the protocol (marked by the dark red rectangle). (*B*) Representative traces in WT, S1021Qfs*98, A228V, and S1021Qfs*98/A228V channels (*n* = 14, 10, 17, and 11, respectively). (C) Voltage dependence of the time constant of inactivation (*τ*_inact_); for significances, see *Table 1*. WT, wild type.

The time constant of inactivation (*τ*_inact_) was studied using protocol which consisted of a 500-ms prepulse from −80 to +50 mV followed by a 2-ms impulse to −120 mV (which enabled full recovery from inactivation and subsequent activation the channel, but avoided its deactivation) and by 500-ms test pulse to voltages between +60 and −60 mV (10-mV steps; the stimulation frequency was 0.2 Hz; for a scheme of the protocol, see *Figure 4A*). The time course of inactivation was fitted using a single exponential function to obtain *τ*_inact_. *Figure 4B* illustrates representative traces from all tested groups. No significant differences compared with WT were observed, but a significant difference was detected between S1021Qfs*98, in which *τ*_inact_ was longer, and A228V as well as S1021Qfs*98/A228V, in which it was shorter (*Figure 4C*, *Table 1*). To be more specific, WT values were between S1021Qfs*98 values, which were ∼56% higher vs. WT, and values in A228V and S1021Qfs*98/A228V, which were ∼37 and 39%, respectively, lower vs. WT.

**Figure 5:**
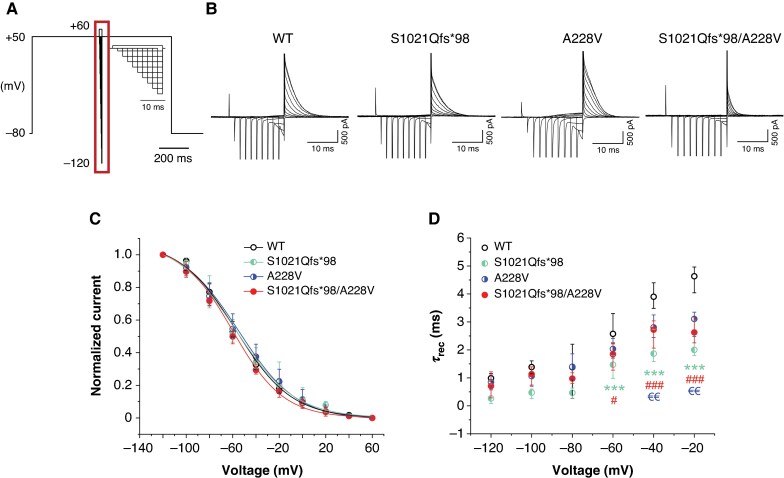
Voltage dependence of steady-state inactivation and recovery from inactivation of Kv11.1 channels. (*A*) Scheme of the stimulation protocol; inset: a detailed view of the key part of the protocol (marked by the dark red rectangle). (*B*) Representative traces in WT, S1021Qfs*98, A228V, and S1021Qfs*98/A228V channels. (*C*) Voltage dependence of steady-state inactivation (*n* = 14, 10, 19, and 17 in WT, S1021Qfs*98, A228V, and S1021Qfs*98/A228V channels, respectively). (*D*) Time constant of recovery from inactivation *τ*_rec_ (*n* = 14, 10, 19, and 15 in WT, S1021Qfs*98, A228V, and S1021Qfs*98/A228V channels, respectively); ***P < 0.001 for S1021Qfs*98 vs. WT; ^€€^*P* < 0.01 for A228V vs. WT; ^#^*P* < 0.05 and ^###^*P* < 0.001 for S1021Qfs*98/A228V vs. WT (Kruskal–Wallis test with Dunn’s post-test; only significances vs. WT are shown, for others, see *Table 1*). WT, wild type.

Voltage dependence of inactivation was studied using a protocol consisting of 500- to 480-ms prepulse from −80 to +50 mV followed by series of short, gradually prolonging impulses to voltages between −120 and +60 mV (the first one to −120 mV lasted only 2 ms, to allow full recovery from inactivation and activation of the channel, but avoid its deactivation, the following impulses were gradually prolonged in 2-ms steps, because all the above-mentioned processes are slower at less negative voltages) and then by 500-ms test pulse to +50 mV followed (the stimulation frequency was 0.2 Hz; *Figure 5A*). Representative traces in all tested groups are shown in *Figure 5B*. The peak current during the test pulse was then evaluated and normalized to its maximal values to obtain the voltage dependence of steady-state inactivation of the channel, which was subsequently fitted using the Boltzmann equation (see Methods). No significant differences in the half-maximal inactivation voltage *V*_1/2_ and slope factor *k* were observed (*Figure 5C*, *Table 1*).

The short and gradually prolonging impulse of the same protocol (the middle one, *Figure 5A*) was used to estimate the time constant of recovery of the channel from inactivation (*τ*_rec_) by fitting with a single exponential function. A significant acceleration of *τ*_rec_ was detected between −60 and −20 mV in S1021Qfs*98/A228V channels vs. WT (e.g. at −20 mV: 4.63 ms in WT, *n* = 14; 1.99 ms in S1021Qfs*98, *n* = 10, *P* < 0.001 vs. WT; 3.10 ms in A228V, *n* = 19, *P* < 0.01 vs. WT; 2.62 ms in S1021Qfs*98/A228V, *n* = 15, *P* < 0.001 vs. WT), whereas no significant differences were observed at more negative voltages (*Figure 5D*, *Table 1*).

To briefly sum up, A228V channels showed minimal or no electrophysiological changes in the Kv11.1 channel function. In contrast, both S1021Qfs*98 and S1021Qfs*98/A228V channels exhibited a markedly reduced current (by 69.5 and 69.2%, respectively, vs. WT) with no changes in the voltage dependence of steady-state activation and inactivation, as well as in the time course of activation and deactivation. Minor alterations were observed in the time course of inactivation and recovery from inactivation.

### Decreased S1021Qfs*98 current results from a reduced Kv11.1 expression

**Figure 6:**
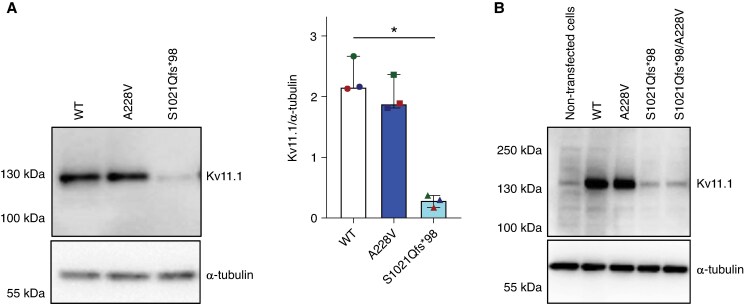
Western blot analysis of Kv11.1 expression after transfection with plasmids for expression of the wild-type (WT) Kv11.1 protein or Kv11.1 protein with the A228V and S1021Qfs*98 variants. (*A*) Representative western blot (for raw original data from three biological replicates, see Supplementary material online, *Figure S3*) and average Kv11.1/α-tubulin values (mean ± IQR); *Statistical significance between WT and S1021Qfs*98 variants at *P* < 0.05 (Kruskal–Wallis test with Dunn’s post-test). (*B*) A substantially diminished Kv11.1 expression was detected in both S1021Qfs*98 and S1021Qfs*98/A228V (ratio 1:1) channels.

The expression of the Kv11.1 protein was studied in CHO cells transiently expressing WT, S1021Qfs*98, and A228V channels using western blot. As illustrated in *Figure 6A*, the expression was substantially reduced in S1021Qfs*98 channels (*P* < 0.05), but it remained unchanged in cells expressing A228V (for raw data from 3 biological replicates, see Supplementary material online, *Figure S3*). When S1021Qfs*98 and A228V variants were coexpressed (1:1), to simulate the situation in the proband, the protein expression was very low, comparable to the expression of the S1021Qfs*98 alone (*Figure 6B*), which agreed well with our observation of substantially reduced current in both S1021Qfs*98 and S1021Qfs*98/A228V channels (*Figure 2B*, *Table 1*). The localization of the S1021Qfs*98 and S1021Qfs*98/A228V channels in the cell membrane was not tested, as the channels exhibited a clear Kv11.1 current (for representative original recordings, see *Figures 2A, 4B,* and *5B*), confirming their presence in the cell membrane.

### Dysfunctional S1021Qfs*98/A228V channels resulted in early afterdepolarization in a human ventricular cell model

**Figure 7:**
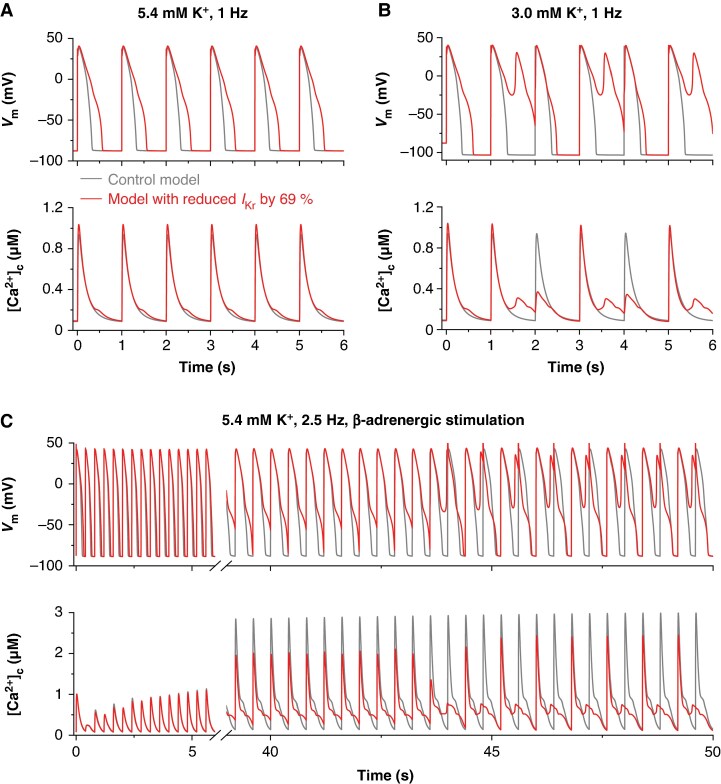
Effects of the *I*_Kr_ reduction induced by S1021Qfs*98/A228V channels (by 69%) on action potential (AP) and cytosolic Ca²⁺ transients (CaT) in a human midmyocardial cell model. (*A*) AP (top) and CaT (bottom) at 1 Hz steady-state stimulation in the control model (grey) and in the model with reduced *I*_Kr_ (red), showing slowed repolarization and prolonged AP duration. (*B*) Response of the models to an acute reduction of [K⁺]ₑ from 5.4 to 3.0 mM at 1 Hz stimulation. The model with reduced *I*_Kr_ persistently generated early afterdepolarizations (EADs) from the second AP, accompanied by irregularities in CaT, whereas the control model remained stable. (*C*) Model behaviour at β-adrenergic stimulation induced by 1 µM isoproterenol (2.5 Hz). After 43 s of regular activity, the model with reduced *I*_Kr_ started to generate persistent EADs.

To simulate the effect of the S1021Qfs*98/A228V variant on the electrophysiological activity of human midmyocardial cells in the O’Hara-Rudy (ORd) model,^13^ we reduced *I*_Kr_ channel conductance (*g*_Kr_) by 69%, consistent with the experimental data. The consequences of this intervention are illustrated in *Figure 7*. As shown in *Figure 7A*, the introduced reduction of *I*_Kr_ markedly slowed AP repolarization, resulting in a pronounced prolongation of APD_90_ (555 ms in the model with reduced *I*_Kr_ vs. 324 ms in the control model) at 1 Hz steady-state stimulation. Considering the appearance of arrhythmia during hypokalemia in the proband, the model response to an acute drop in the extracellular K⁺ concentration ([K⁺]ₑ) from 5.4 to 3.0 mM during 1 Hz stimulation was subsequently simulated. In contrast to the control model, the model with reduced *I*_Kr_ began to generate persistent EADs from the second AP after the reduction of [K⁺]ₑ, which was accompanied by irregularities in CaT (*Figure 7B*). Since the proband also experienced arrhythmias during abrupt stress (e.g. when being suddenly scared or during physical activity), we performed additional simulations under β-AS induced by 1 µM isoproterenol (*Figure 7C*). When β-AS was initiated and the stimulation frequency was increased from 1 to 2.5 Hz, the model with reduced *I*_Kr_ began to generate EADs after approximately 43 s. These EADs persisted in a sustained pattern thereafter. Besides *I*_Ca_ reactivation as the primary EAD trigger, the inward mode of the Na⁺-Ca²⁺ exchanger current (*I*_NaCa_) represented an additional important factor supporting EAD formation (for details, see Discussion).

Taken together, the simulations demonstrated that both reduced [K^+^]_e_ and an abrupt stress represented important proarrhythmic factors in carriers of the S1021Qfs*98 variant.

## Discussion

This study reports clinical, genetic, and functional characteristics of a compound heterozygosity in the *KCNH2* (*hERG*) gene, namely S1021Qfs*98 and A228V variants, in a proband suffering from idiopathic VF. A complex functional analysis revealed that the S1021Qfs*98 variant is a loss-of-function variant with a dominant-negative effect in coexpression with the second, benign A228V variant, resulting in a marked decrease in the channel expression and function in S1021Qfs*98 as well as S1021Qfs*98/A228V channels, the latter mimicking the channels of the proband. This dysfunction resulted in EADs in a human ventricular cell model, which suggests arrhythmogenic potential of the S1021Qfs*98 variant.

### Truncating variants in the C-terminus of the Kv11.1 protein

The C-terminus of the Kv11.1 protein, encoded by the *KCNH2* gene, contains several critical regulatory domains, responsible for modulation of *I*_Kr_ inactivation, protein trafficking, and subunit oligomerization.^20,21^ That corresponds with the loss-of-function character of the distal C-terminal, arrhythmogenic variant S1021Qfs*98, caused by the premature stop codon and reduced expression of the channel.

The loss-of-function character has been documented in other truncating *KCNH2* variants, usually resulting in symptoms of LQT2 in carriers.^20-25^ Similarly to our study, some of these variants impaired the expression/trafficking of the channels, showing the dominant negativity as well (e.g. S981Lfs*135, A915Rfs*48, Q725*, and R1014*).^22,23,25^ This is likely related to the consequent deletion or disruption of the so-called tetramerizing coiled-coil, which is a part of the distal C-terminus of the channel and is known to be essential for proper channel assembly.^21,25^ In other C-terminal truncating *KCNH2* variants, the expression/trafficking was normal, but the gating properties of the channels were defective. This resulted in a rightward activation shift as well as faster kinetics of recovery from inactivation (G1006Afs*51)^20^ and inactivation (G1006Afs*51, P923Rfs*51).^20,26^ Interestingly, Paulussen *et al.*^24^ described the G873Afs*5 variant with a current similar to WT channels when expressed separately, but showing the gain-of-function defect in gating properties (demonstrated by AP voltage clamp experiments) as well as loss-of-function due to a trafficking deficiency and dominant-negative effect when coexpressed with WT; the final dysfunction resulted in delayed repolarization and LQT2. In some studies, the functional analysis is missing,^27^ which disallows the definitive conclusion on the variant’s pathogenic potential, despite being classified as pathogenic by ACMG guidelines.

### *In silico* insights into arrhythmogenesis induced by S1021Qfs*98/A228V

Electrophysiological consequences of the experimentally observed S1021Qfs*98/A228V dysfunction in the Kv11.1 (*I*_Kr_) channels were investigated using the ORd human ventricular cell model.^13^ The variant caused a ∼69% reduction of *I*_Kr_, while no or only subtle changes were detected in voltage- and time-dependent characteristics of the channel gating. Hence, a proportional reduction in *g*_Kr_ was the only physiologically justified intervention in the used ORd model. Within this framework, the model predicted APD_90_ prolongation by 71% in control conditions (resting heart rate, normokalemia), and the formation of EADs under hypokalemic conditions (resting heart rate, 3.0 mM) as well as during β-AS accompanied by an increase in stimulation frequency to 2.5 Hz (*Figure 7*).

EAD formation was driven primarily by *I*_Ca_ reactivation facilitated by slowed repolarization, a consequence of the *I*_Kr_ reduction. However, an increased activity of the inward *I*_NaCa_ represented an additional depolarizing factor driving EADs. This was a consequence of the elevated cytosolic Ca²⁺ levels (as visible in *Figure 7C* before the onset of EADs, 39–43 s) produced by the premature Ca²⁺ release triggered by the above-mentioned *I*_Ca_ reactivation. The premature Ca²⁺ release also resulted in a reduced Ca²⁺ content in the junctional sarcoplasmic reticulum, which explains the lower CaT amplitude during β-AS at 2.5 Hz in the model with reduced *I*_Kr_ vs. the control model (*Figure 7C*).

These computational findings are qualitatively consistent with the clinical phenotype of the proband carrying the S1021Qfs*98/A228V variant, who exhibited arrhythmias mainly under hypokalemia (2.9 mM) and during stress conditions. These results highlight the critical role of the genetic defect and environmental factors, such as potassium imbalance and stress, in the pathogenesis of arrhythmias, which is in line with previously published studies.^28–32^

### *KCNH2* variants associated with idiopathic VF or low-penetrant long QT syndrome type 2

In some patients, variants in genes encoding cardiac ionic channels or associated proteins do not result in a typical phenotype. Consequently, a definitive diagnosis of a particular inherited arrhythmogenic syndrome cannot be made and the diagnosis of idiopathic VF is suggested (for a retrospective overview, see Verheul *et al.*^5^). It also applies to loss-of-function variants in the *KCNH2* gene, which are usually associated with the congenital LQT2 (for a recent review, see Ponce-Balbuena and Deschênes^33^). The clinical diagnosis of LQT2 is primarily based on a characteristic phenotype of LQT2, quantified by the diagnostic score.^10,11^ In contrast, idiopathic VF is a diagnosis *per exclusionem*, when characteristic features of LQT2 (or other specific inherited arrhythmogenic syndrome) are absent.^34^ In some cases, the original diagnosis of idiopathic VF can be changed after a deep investigation, as visible *e.g.* in the study by Jiménez-Jáimez *et al.*^35^ These authors reported four patients with the initial diagnosis of IVF, from whom one, having a baseline QTc 405 ms, was reclassified to LQT2 after further clinical examination (electrophysiological study, coronary angiography, and flecainide and epinephrine challenge) and genetic testing (three variants in the *KCNH2* gene). Unfortunately, the functional analysis was not performed in that study; thus, the pathogenetic mechanism is still unclear.

Some variants in the *KCNH2* gene not clearly related to congenital LQT2 have already been functionally characterized, including the C-terminal variant S906L.^6^ This variant was found in five male and three female members of a Dutch family. Despite carrying the same variant, the QTc interval in the studied patients ranged from normal to prolonged, with variable severity of the phenotype. The variant was later classified as a low-penetrant, likely pathogenic variant, as a decreased current density and a decelerated time course of activation were identified during the functional analysis. Varying penetrance was also observed in the variant P347S,^7^ which was classified as pathogenic based on functional testing (faster inactivation kinetics, but slower kinetics of recovery from inactivation combined with a dominant-negative reduction of the current due to impaired trafficking when co-transfected with WT). In some carriers of this variant, the diagnosis of LQT2 was made, but others were asymptomatic, or the prolonged QT interval was observed only after concurrent administration of multiple QT-prolonging drugs. These studies emphasize the importance of functional analysis in the final decision on the pathogenicity of an identified genetic variant, as well as in establishing a definitive clinical diagnosis, especially in variants with incomplete penetrance.

In the case of our proband, the diagnosis of idiopathic VF was set at the time of the first VF episode, more than two decades ago, since the QTc values of this female proband have never exceeded 460 ms. Interestingly, her syncopal events were often related to emotional stress (such as the alarm clock ringing), which is considered a typical feature of the LQT2 phenotype. Despite this atypical clinical picture, the functional analysis and *in silico* simulations performed in this study revealed that the S1021Qfs*98 variant (classified as a likely pathogenic variant) resulted in a reduced expression of the channels and a markedly decreased current, leading to a significant repolarization delay and EADs. In the meantime, the proband gave birth to a daughter, who also carries the S1021Qfs*98 variant and developed symptoms of LQT2. With this ‘Family member with definitive LQTS’ and functional confirmation of the variant pathogenicity, the proband can finally be diagnosed as LQT2. Moreover, the 2022 ESC Guidelines for the management of patients with ventricular arrhythmias presented a new version of the LQTS diagnostic score, where the finding of a pathogenic variant alone is sufficient for the LQTS diagnosis.^8,9^ Nevertheless, this is somewhat in controversy with another scientific document—the 2022 consensus statement on genetic testing for cardiac diseases, according to which it is appropriate to perform genetic testing in patients with a specific phenotype.^36^ Our case could be considered as an example when even an ‘unclear’ phenotype justifies the genetic testing, as it could lead to a confirmation of the LQTS diagnosis and could enable appropriate risk stratification and therapy.

### Limitations of the study

The dysfunction in the variants S1021Qfs*98 and A228V coexpressed with WT subunits was not tested. Since A228V generated a current comparable to WT (*Figure 2B*, *Table 1*), the coexpression of A228V and WT subunits would very likely not show any differences compared with the expression of WT alone. Considering almost identical currents in S1021Qfs*98 and S1021Qfs*98/A228V channels (*Figure 2B*, *Table 1*) and comparably decreased expression in these channels (*Figure 6B*), which both support our idea that S1021Qfs*98 is a dominant negative variant, we expect that an analogous dysfunction, that we can see in S1021Qfs*98/A228V channels (*Figure 2B*), would be present in S1021Qfs*98/WT channels.

The use of CHO cells as the model organism expressing the human Kv11.1 channels may also represent a limitation of this study. Only a single Kv11.1-positive band can be identified in CHO cells in our study (*Figure 6*) as well as in some previously published studies,^37,38^ but in contrast to *e.g.* the study by Asahi *et al.*^39^ The missing matured band might be due to, e.g., a limited ability of CHO cells to glycosylate the Kv11.1 protein or by specificity of the used antibody to the glycosylated form. Nevertheless, the suitability of using CHO cells is consistent with many previously published works that have used CHO cells in a similar context, including studies of the Kv11.1 protein function.^37,38,40,41^

There is an apparent discrepancy between the absence of QT prolongation in the proband carrying the S1021Qfs*98/A228V variant in the Kv11.1 channel (*Figure 1C*) and the marked APD prolongation predicted by the *in silico* simulations using the ORd human ventricular cell model (*Figure 7A*). In contrast, QT prolongation has been observed in the proband’s daughter, who also carries the pathological S1021Qfs*98 variant. We hypothesize that this discrepancy may reflect individual electrophysiological differences that could attenuate the QT-prolonging effect of the loss-of-function S1021Qfs*98 variant in the proband. Future studies using patient-specific cellular or tissue models may help to clarify these inter-individual differences further.

While the presented single-cell simulations provide a mechanistic explanation for the electrophysiological abnormalities observed in the proband carrying the S1021Qfs*98/A228V variant, the model outcomes should be interpreted qualitatively rather than quantitatively. More comprehensive approaches, such as 2D or 3D cardiac tissue models, integrating the effects of *I*_Kr_ reduction with electrotonic coupling, regional heterogeneity of repolarization, and autonomic influences, may capture the variant-related phenotype with greater fidelity. Such multiscale models could better reflect how cellular dysfunction translates into the whole-heart manifestation of arrhythmogenesis.

### Conclusions

Based on clinical, genetic, and functional analysis of our compound heterozygous *KCNH2* gene proband, we conclude that the A228V variant is benign, but the S1021Qfs*98 variant is pathogenic, substantially increasing arrhythmogenicity, especially during hypokalemia and abrupt stress. Hence, the latter variant is likely responsible for the clinical symptoms observed in the proband. Despite the absence of a prolonged QT interval in the available ECG records of the proband, the detailed functional analysis of the S1021Qfs*98 variant performed *in vitro* and *in silico* within this study has enabled the reclassification of the clinical diagnosis from idiopathic VF to LQT2. This study demonstrates the necessity of functional analysis for final clinical diagnosis and adequate management of patients with idiopathic VF and variants in cardiac ionic channels genes or associated proteins.

## Supplementary Material

euag001_Supplementary_Data

## Data Availability

The relevant data are available from the corresponding author upon reasonable request.
