# Supplementary material for: Identification and functional assessment of a KCNH2 compound heterozygosity in a patient with presumed idiopathic ventricular fibrillation ascertains the diagnosis of long QT syndrome type 2
Source: Europace. 2026 Jan 9;28(2):euag001. doi: 10.1093/europace/euag001 (PMC12950810; doi:10.1093/europace/euag001)
Supplement: euag001_Supplementary_Data [file euag001_supplementary_data.zip › Supplementary Figure S1.pdf]

Supplementary Figure S1

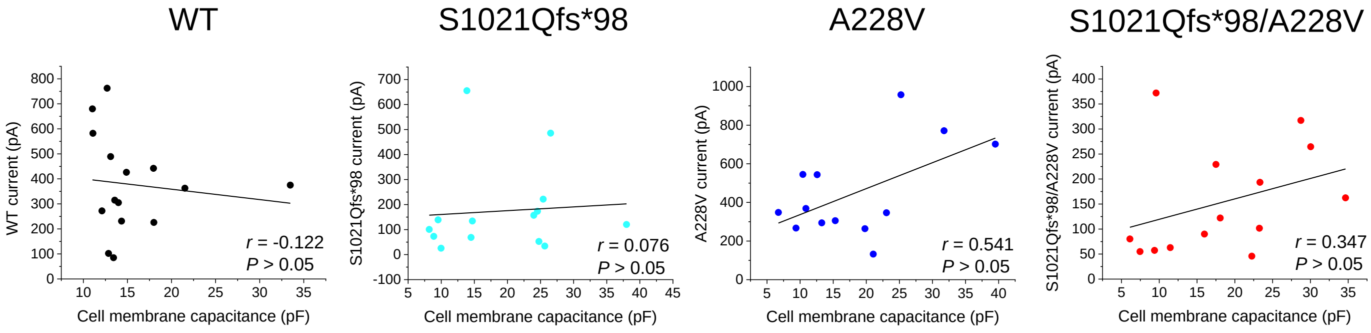

Supplementary Figure S1: Relationship between the current amplitude and cell membrane capacitance. Since a significant positive correlation was not identified in any tested cell groups, conversion of the current amplitude to the current density was not performed in this study.
