# Supplementary material for: Identification and functional assessment of a KCNH2 compound heterozygosity in a patient with presumed idiopathic ventricular fibrillation ascertains the diagnosis of long QT syndrome type 2
Source: Europace. 2026 Jan 9;28(2):euag001. doi: 10.1093/europace/euag001 (PMC12950810; doi:10.1093/europace/euag001)
Supplement: euag001_Supplementary_Data [file euag001_supplementary_data.zip › Supplementary Figure S3.pdf]

Supplementary Figure S3

A

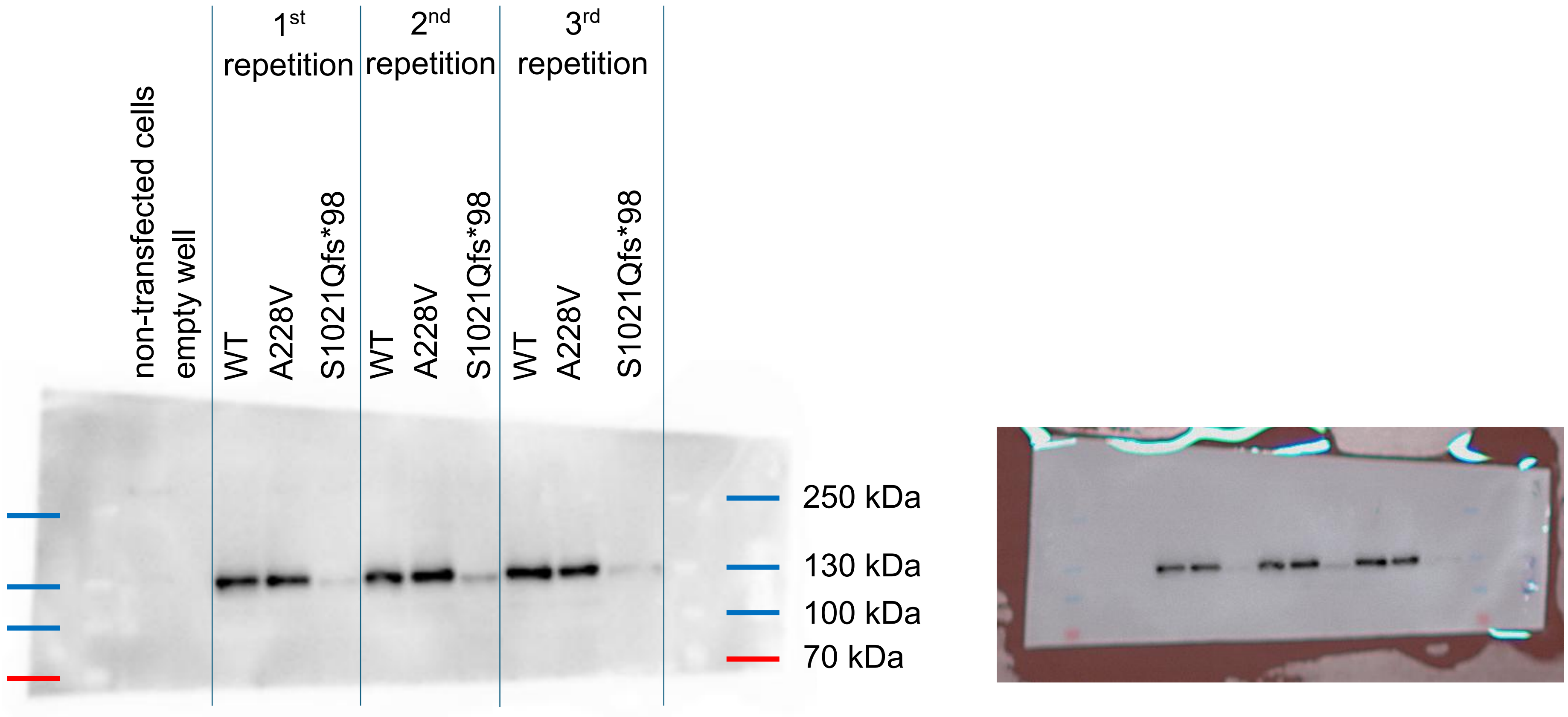

Supplementary Figure S3-A: Western blot analysis of Kv11.1 expression after transfection with plasmids for expression of wild type (WT) or mutant (A228V and S1021Qfs*98) variants of the protein. **A:** Raw original data from individual replicates – Kv11.1 (exposition 30 s).

Supplementary Figure S3

B

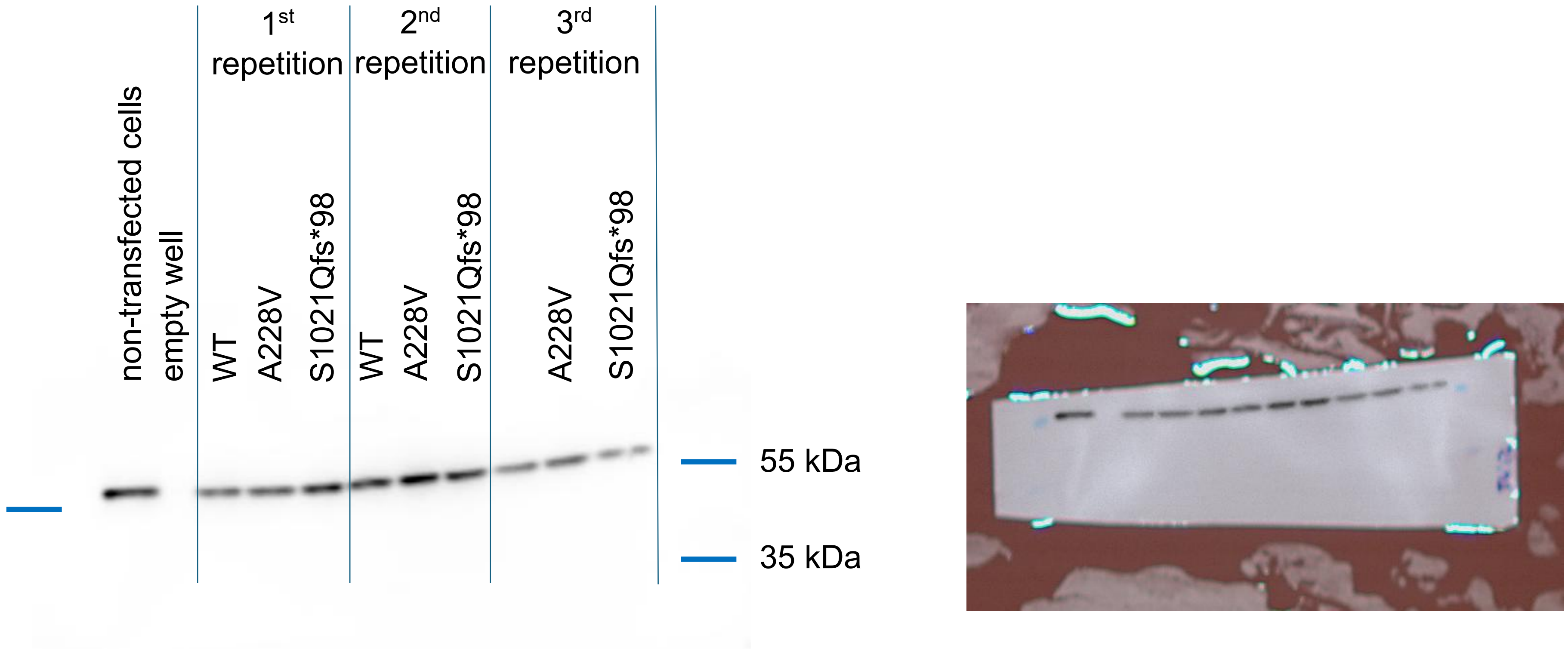

Supplementary Figure S3-B: Western blot analysis of Kv11.1 expression after transfection with plasmids for expression of wild type (WT) or mutant (A228V and S1021Qfs*98) variants of the protein. **B:** Raw original data from individual replicates – α-tubulin (exposition 5 s).
